# A Dozen Hardy Shrubs

**Published:** 1885-06

**Authors:** 


### A Dozen Hardy Shrubs.

To an inquirer in the *Rural New-Yorker* for the names of a dozen of the best ornamental flowering shrubs, Mr. C. E. Parnell, of Queens, L. I., replies as follows :

It is really a difficult affair to select a dozen only, for there are so many beautiful sorts, and all of them present so many claims to our notice, that it appears to be altogether unjust to neglect the many on account of a few. But as there are many who, like your correspondent, only desire, or have room for a few, one cannot do less than make the attempt at a selection. First, I would choose Weigela nana variegata, one of the most beautiful shrubs in cultivation. It is of dwarf habit, with clearly defined variegated leaves of a bright golden yellow. The flowers, which are of a pale rose color, are produced in the greatest profusion early in June. Weigela rosea Desboisii is of erect, compact growth, and has deep rose-colored flowers in June. Spiræa Thunbergii is a beautiful low-growing shrub of rounded form, and has delicate green lanceolate foliage, and small white flowers, which are produced early in May in such profusion as almost to cover the entire plant. Spiræa Reevesiana is a very graceful, slightly drooping species, with white flowers ; while S. callosa alba is a low-growing variety, producing its small, white flowers in large corymbs during June and July. Philadelphus coronarius is rather a long name for a very popular and well-known strong growing shrub that produces its large, pure white, sweet-scented flowers about the middle of June. Hydrangea Paniculata grandiflora is so well known as to need no further description than to say that it is one of the best, if not the best, ornamental shrub we have in cultivation. Buist's Variegated Althæa is another choice variegated shrub, the leaves of which are beautifully marked with creamy white. It stands the sun well, is of free growth, and is attractive at all times. Then we must include the Golden Bell (Forsythia viridissima), which is well known as one of the earliest flowering shrubs, the bright yellow flowers appearing before the leaves. Deutzia crenata fl pl. alba produces its double, white flowers in racemes four or five inches in length late in June, and is a shrub of vigorous growth ; while D. Gracilis is one of the most graceful of shrubs. It is of dwarf, compact habit, and the pure white flowers are most freely produced. The Persian Lilac (Syringa Persica) is a shrub of medium size, having small leaves and purple, fragrant flowers.

All of the above are perfectly hardy, and can be cultivated by anyone, even by those who possess but little skill or experience, and, if properly cared for, they will prove very satisfactory. They are not rare or expensive, and nice specimens can be obtained at a very moderate price of any of our leading nurserymen.

